# Matricellular Protein SMOC2 Potentiates BMP9-Induced Osteogenic Differentiation in Mesenchymal Stem Cells through the Enhancement of FAK/PI3K/AKT Signaling

**DOI:** 10.1155/2023/5915988

**Published:** 2023-01-16

**Authors:** Wen-Ge He, Yi-Xuan Deng, Kai-Xin Ke, Xuan-Lin Cao, Si-Yuan Liu, Yuan-Yuan Yang, Hong-Hong Luo, Xin-Tong Yao, Xiang Gao, Yu Du, Bai-cheng He, Liang Chen

**Affiliations:** ^1^Department of Bone and Soft Tissue Oncology, Chongqing University Cancer Hospital, Chongqing 400030, China; ^2^Department of Orthopedics, The Second Affiliated Hospital, Chongqing Medical University, Chongqing 400010, China; ^3^Key Laboratory of Biochemistry and Molecular Pharmacology of Chongqing, Chongqing Medical University, Chongqing 400016, China; ^4^Department of Pharmacology, School of Pharmacy, Chongqing Medical University, Chongqing 400016, China

## Abstract

Mesenchymal stem cells (MSCs) can self-renew and differentiate into multiple lineages, making MSC transplantation a promising option for bone regeneration. Both matricellular proteins and growth factors play an important role in regulating stem cell fate. In this study, we investigated the effects of matricellular protein SMOC2 (secreted modular calcium-binding protein 2) on bone morphogenetic protein 9 (BMP9) in mouse embryonic fibroblasts (MEFs) and revealed a possible molecular mechanism underlying this process. We found that SMOC2 was detectable in MEFs and that exogenous SMOC2 expression potentiated BMP9-induced osteogenic markers, matrix mineralization, and ectopic bone formation, whereas SMOC2 knockdown inhibited these effects. BMP9 increased the levels of p-FAK and p-AKT, which were either enhanced or reduced by SMOC2 and FAK silencing, respectively. BMP9-induced osteogenic markers were increased by SMOC2, and this increase was partially abolished by silencing FAK or LY290042. Furthermore, we found that general transcription factor 2I (GTF2I) was enriched at the promoter region of SMOC2 and that integrin *β*1 interacted with SMOC2 in BMP9-treated MEFs. Our findings demonstrate that SMOC2 can promote BMP9-induced osteogenic differentiation by enhancing the FAK/PI3K/AKT pathway, which may be triggered by facilitating the interaction between SMOC2 and integrin *β*1.

## 1. Introduction

BMP9 plays an important role in stem cell fate regulation [1]. It can induce mesenchymal stem cell (MSC) differentiation into osteoblasts, which has promising clinical applications [2]. BMP9 exerts physiological effects in stem cell biology through classical and/or nonclassical BMP/Smad signaling, such as p38 MAPK and PI3K/Akt [3, 4]. However, due to some limitations, such as longer maturation times for bone matrix and uncontrolled adipogenesis differentiation, clinical applications of BMP9 are still a long way off [5]. As a result, to improve the chances of the clinical application of BMP9, the osteogenic capacity of BMP9 needs to be enhanced, and the potential mechanisms of BMP9-induced osteogenesis need to be elucidated.

Secreted modular calcium-binding protein 2 (SMOC2) is a matricellular protein, which belongs to the SPARC-related modular calcium-binding protein family, that has two isoforms. The SPARC family is well known for its involvement in bone mineralization, cell-matrix interactions, collagen binding, and bone remodeling [6–8]. SMOC1, another isoform of SMOCs, has been found to be substantially expressed in bone marrow mesenchymal stem cells. Overexpression of SMOC1 promotes osteoblast differentiation, whereas knockdown of SMOC1 inhibits osteoblast differentiation and mineralization [9]. However, the mechanisms by which SMOC2 affects the skeletal bone system are controversial. Mutant SMOC2 was shown to inhibit BMP signaling by competitively binding to BMPR1B, which resulted in growth plate defects and short-limb dwarfism. This finding indicates an important role of SMOC2 in bone development [10]. In contrast, SMOC2 was also reported to inhibit osteogenic differentiation and extracellular matrix mineralization in MC3T3-E1 cells through the SMOC2 EC domain only [11]. These differential effects of SMOC2 may be caused by several factors, including the cell types and the cellular microenvironment. However, the effect of SMOC2 on the osteogenic activity induced by BMP9 remains unclear; moreover, the relationship between BMP9 and SMOC2 in progenitor cells during the process of osteogenic commitment requires further study.

FAK is a nonreceptor tyrosine kinase found in the cytoplasm that, like other kinases, carries out cellular signaling by phosphorylating downstream substrates [12]. FAK can cross-talk with multiple signal transduction pathways to regulate cell survival, proliferation, motility, and differentiation through its kinase-independent and kinase-dependent functions in different cellular processes [13–16]. FAK is a key regulator in integrin-mediated signaling pathways [17] and is phosphorylated in response to integrin involvement, activating downstream signals. FAK has been shown to promote osteogenesis by enhancing Wnt-*β*-catenin signaling [18]. However, the precise mechanism underlying the function of FAK in the osteogenic process of MSCs needs further investigation.

In the present study, we introduced a series of in vitro and in vivo experiments to study the effect of SMOC2 on BMP9-induced osteogenic differentiation in MSCs and verified a possible mechanism underlying this effect. Our findings may provide another efficacious strategy to enhance BMP9-induced osteogenesis in MSCs and broaden the role of matricellular proteins in regulating stem cell fate.

## 2. Materials and Methods

### 2.1. Cell Culture and Chemicals

HEK293 cells were purchased from ATCC (VA, USA). Cells were maintained in complete Dulbecco's modified Eagle's medium (DMEM) with 10% fetal bovine serum (FBS), 100 U/ml penicillin, and 100 ug/ml streptomycin at 37°C in 5% CO_2_. Primary antibodies against *β*-actin (sc-47778), OPN (sc-21742), and SMOC2 (sc-376104) were purchased from Santa Cruz Biotechnology (Shanghai, China). Primary antibodies against RUNX2 (12556S), p-AKT1/2/3 (4060S), and AKT1/2/3 (9272) were purchased from Cell Signaling Technology (Shanghai, China). A primary antibody against FAK (66258-1-Ig) was purchased from Proteintech. A primary antibody against p-FAK (ab81298) was ordered from Abcam, and the PI3K inhibitor LY294002 was purchased from MCE (USA).

### 2.2. Recombinant Adenovirus Construction

Recombinant adenoviruses were generated using AdEasy technology as previously described [19, 20]. The coding sequences of mouse BMP9 and SMOC2 were amplified using PCR. The PCR products and the siRNA oligo fragments for SMOC2 were all subcloned into adenoviral shuttle vectors. Then, these vectors were used to generate recombinant adenoviruses in HEK293 cells. The recombinant adenoviruses were designated AdBMP9, AdSMOC2, and AdsiSMOC2. To track the viruses, AdBMP9 and AdSMOC2 were tagged with green fluorescent protein (GFP), and AdsiSMOC2 was tagged with red fluorescent protein (RFP). Analogous adenoviruses expressing only monomeric RFP (AdRFP) or GFP (AdGFP) were used as controls.

### 2.3. Isolation of Mouse Embryonic Fibroblasts (MEFs)

As previously described, MEFs were isolated from mice on postcoitus Day 12.5. Each embryo was dissected into 10 ml of sterile PBS, voided of its internal organs, and sheared through an 18-gauge syringe in the presence of 1 ml of 0.25% trypsin with 1 mM EDTA. After 15 minutes of gentle shaking at 37°C, 10 ml DMEM with 10% FBS was added to deactivate the trypsin. The cells were plated on 100 mm dishes and incubated for 24 hours at 37°C before being used as MEFs. The MEFs within 5 passages were used in this study.

### 2.4. RNA Extraction, cDNA Preparation, and Quantitative Polymerase Chain Reaction (Q-PCR) Analysis

Total RNA was extracted from cells using TRIzol reagent (Invitrogen, USA), purified, and used to generate cDNA via reverse transcription (RT). SYBR green mixture kits were used for real-time PCR using the Bio-Rad CFX Connect system. The target mRNA levels were normalized to the expression of *β*-actin.  Table 1 shows the PCR primer sequences used in this study.

### 2.5. Protein Harvesting and Western Blotting

Cells were harvested on ice in PBS and centrifuged at the designated time point. RIPA lysis solution (R0020, Solarbio, China) (300 *μ*l) was added to each 1.5 ml centrifuge tube, and the lysates were denatured by boiling for 10 minutes. Protein samples were separated using a 10% SDS–PAGE gel. The proteins were then transferred to a polyvinylidene difluoride (PVDF) membrane, which was blocked in 5% skimmed milk for 2 hours and then incubated overnight at 4°C with diluted antibodies. Tris-buffered saline Tween 20 (TBST) was used to dissolve bovine serum albumin (BSA), and the antibodies were diluted with 3% BSA. TBST was used to wash the membranes three times. The membranes were then incubated with secondary antibodies for 1 hour at room temperature. The membranes were then washed three times with TBST before being visualized with an HRP chemiluminescent substrate reagent kit and the data were collected and quantitatively analyzed using the Bio-Rad Chemx system (Bio-Rad, USA).

### 2.6. Alkaline Phosphatase (ALP) Assay

Cells were seeded on 24-well plates and then treated with a variety of factors before being incubated for 5 or 7 days. The cells in the 24-well plates were then washed twice with PBS before being stained with the Alkaline Phosphatase Assay Kit (C3206, Beyotime, Jiangsu, China). In summary, ALP staining solution was added, and the cells were left at room temperature for 15 minutes without exposure to light. Finally, the data were gathered by scanning the plates. These findings were replicated in at least three separate experiments.

### 2.7. Matrix Mineralization Assay (Alizarin Red S Staining)

MEFs were seeded on 24-well plates and treated with the designated factors. Cells were cultured in the presence of dexamethasone (10 nM), ascorbic acid (50 *μ*g/ml), and *β*-glycerophosphate (10 mM). Mineralized matrix nodules were stained with Alizarin Red S 14 or 21 days after being cultured, as previously described. The cells were fixed with 0.05% (*v*/*v*) glutaraldehyde for 10 minutes at room temperature. After washing with distilled water (pH 4.2), the fixed cells were treated with 0.4% Alizarin Red S (Sigma–Aldrich) for 15 minutes before being scanned and/or photographed under a microscope.

### 2.8. Immunofluorescence Staining Assay

The cells were seeded in 48-well plates and treated with the appropriate factors before being incubated at 37°C for 24 hours. The cells were then washed twice with PBS and fixed for 10 minutes in 4% paraformaldehyde. Subsequently, they were incubated in 0.3% Triton X-100 (Solarbio, Beijing, China) to increase the permeability of the cell membrane, followed by three washes with PBS. They were then blocked with goat serum (Beyotime, Shanghai, China) at room temperature for 1 hour before incubation with the target antibodies overnight at 4°C. The antibodies were recycled the following day, and the cells were washed three times with PBS before being incubated in the dark with DyLight 594-conjugated secondary antibody at 37°C. The cells were then washed twice with PBS and stained for 5 minutes with 4,6-diamino-2-phenyl indole (DAPI). Finally, they were washed three times with PBS and immersed in 80% glycerin to obtain the immunofluorescent images. The dilution ratios were 1 : 200 for the primary antibodies, 1 : 100 for the secondary antibody, and 1 : 10,000 for DAPI. These findings were replicated in at least three independent experiments.

### 2.9. Ectopic Bone Formation

Four-week-old nude female mice (*n* = 5/group) were used for these experiments. Nude mice were ordered from Hua Fu Kang Biotechnology Co., Ltd. (Beijing, China). This study was approved by the Institutional Animal Care and Use Committee of Chongqing Medical University. The MEFs were first grown in 100-mm dishes. According to the experimental design, the cells were injected subcutaneously into athymic nude mice twenty-four hours after treatment (5 × 10^6^ cells per injection). After four weeks, all of the animals were euthanized, and bone samples were collected and treated in 10% formalin.

### 2.10. Histological Staining and Evaluation

Ectopic bone samples were fixed with 4% paraformaldehyde for three days, decalcified with EDTA solution (pH 7.2) for four weeks, and then embedded in paraffin. The paraffin slices were stained with hematoxylin and eosin (H&E), Masson trichrome, and immunohistochemical reagents after deparaffinization and rehydration.

### 2.11. Chromatin Immunoprecipitation (ChIP) Assay

Standard ChIP analysis was performed as previously described [21] 30 hours following AdGFP or AdBMP9 infection. An antibody against general transcription factor 2 I (GTF2I) was used to precipitate the protein–DNA complexes; rabbit IgG was utilized as a reference. The enrichment of SMOC2 promoter fragments was next investigated using a PCR assay. The primer sequences for this assay are listed in Table 1.

### 2.12. Immunoprecipitation (IP)

Cells were lysed 30 hours after infection with RIPA lysis buffer (R0020, Solarbio, China) containing protease and phosphatase inhibitors (B14002 and B15002, Bimake, Shanghai, China); all procedures were carried out on ice. Protein G magnetic beads (S1430, NEB China group, Beijing, China) were prewashed with 30 *μ*L lysis buffer. Cell lysates were incubated with the SMOC2 antibody for 10 hours at 4°C. After that, 15 *μ*L of prewashed protein G magnetic beads was added to the lysates and incubated at 4°C for 1 hour. A magnetic stand was used to collect the target complexes, which were then washed with lysis buffer. The proteins were then eluted from the beads using 30 *μ*L of lysis buffer.

### 2.13. Statistical Analysis

The quantitative data are presented as the mean ± SD. For comparisons between two groups, the two-tailed Student's *t* test was utilized. Each assay condition was performed in triplicate for all quantitative assays, and the results were replicated in at least three independent experiments. All data collected were statistically analyzed. A p value of less than 0.05 was defined as statistically significant.

## 3. Results

### 3.1. BMP9 Upregulates the Expression of SMOC2 in MSCs

**Figure 1:**
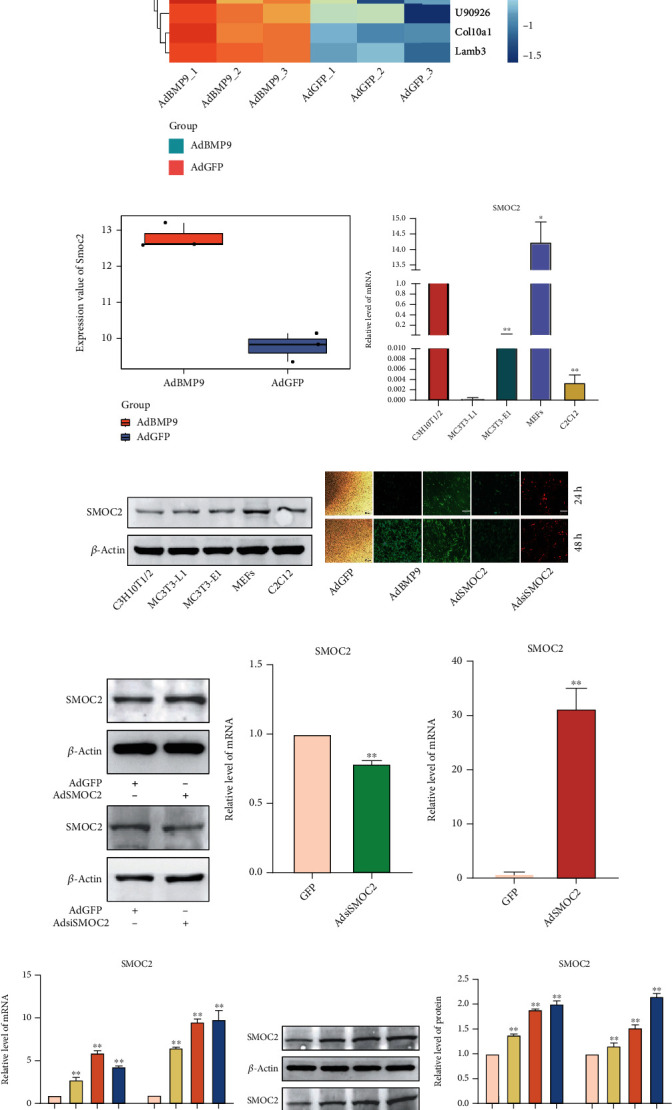
BMP9 upregulates the expression of SMOC2 in MSCs. (a) Heatmap of the top 10 upregulated and downregulated mRNAs between the control (AdGFP) and experimental groups (AdBMP9). (b) RNA-seq analysis showed the expression value of *s*moc2 between AdGFP and AdBMP9. (c) Real-time PCR assay results showed endogenous mRNA expression of *s*moc2 in mesenchymal progenitor cells. (d) Western blot assay results showed endogenous protein expression in mesenchymal progenitor cells; *β*-actin was used as an internal reference. (e) Efficient transduction of MEFs by the recombinant adenoviruses (AdGFP, AdBMP9, AdSMOC2, and AdsiSMOC2) at 24 h. The expression of the marker genes monomeric RFP and GFP was detected at 24 h after infection in both bright and fluorescence fields. (f–h) Western blot and real-time PCR assays showed the expression of SMOC2 in MEFs at 24 h after infection by recombinant adenoviruses overexpressing SMOC2 and knocking down SMOC2. (i) Real-time PCR assay results showed the effect of BMP9 on mRNA expression of *s*moc2 in MEFs (^∗^*p* < 0.05, ^∗∗^*p* < 0.01 vs. control). (j) Western blot assays showed the effect of BMP9 on SMOC2 protein expression in MEFs. (k) The quantification results of the Western blot assay showed the effect of BMP9 on the protein expression of SMOC2 in MEFs (^∗^*p* < 0.05, ^∗∗^*p* < 0.01 vs. control).

First, to investigate which genes are differentially expressed in BMP9-induced osteogenic differentiation of MEFs, we performed RNA-seq analysis on the control (AdGFP) and experimental groups (AdBMP9) at 24 h, and to ensure that the experiment was statistically significant, three samples were assessed in each group. Based on the results of the presequencing samples, genes with adj. *p* values less than 0.05 and |log_2_FC| greater than 1 were considered as DEGs by using the LIMMA package in R. Among all the differentially expressed genes, SMOC2 was more highly expressed in the experimental groups than in the control groups (Figures 1(a) and 1(b)). These results indicate that SMOC2 may act as a downstream target of BMP9. Since the role of matricellular proteins, in particular the specific proteins, in the osteodifferentiation of stem cells is unclear, the detailed mechanism of SMOC2 function needs to be further studied. Real-time PCR analysis and western blotting results showed that the expression of SMOC2 was detectable in several progenitor cells, and we found that the endogenous expression of SMOC2 was the highest in the MEFs that we used in the current investigation (Figures 1(c) and 1(d)). While the detailed regulatory mechanism remains to be fully investigated, these results indicate that SMOC2 plays an important role in BMP9-induced osteogenic differentiation of MEFs.

To further investigate the role of SMOC2 in BMP9 signaling, we constructed and generated a recombinant adenovirus expressing mouse SMOC2 using the AdEasy system. As shown in Figure 1(e), the generated adenoviruses transduced MEFs with high efficiency. Real-time PCR analysis and western blotting demonstrated that the overexpressing adenovirus (AdSMOC2) and silencing adenovirus (AdsiSMOC2) were effectively constructed (Figures 1(f)–1(h)). In MEFs, our real-time PCR and western blotting results indicated that BMP9 could obviously induce SMOC2 expression, which is consistent with the RNA-seq analysis (Figures 1(i)–1(k)). These data suggest that SMOC2 may be associated with the process of osteogenic lineage commitment in MSCs via BMP9 stimulation.

### 3.2. The Effect of SMOC2 on BMP9-Induced Early Osteogenic Marker Levels

**Figure 2:**
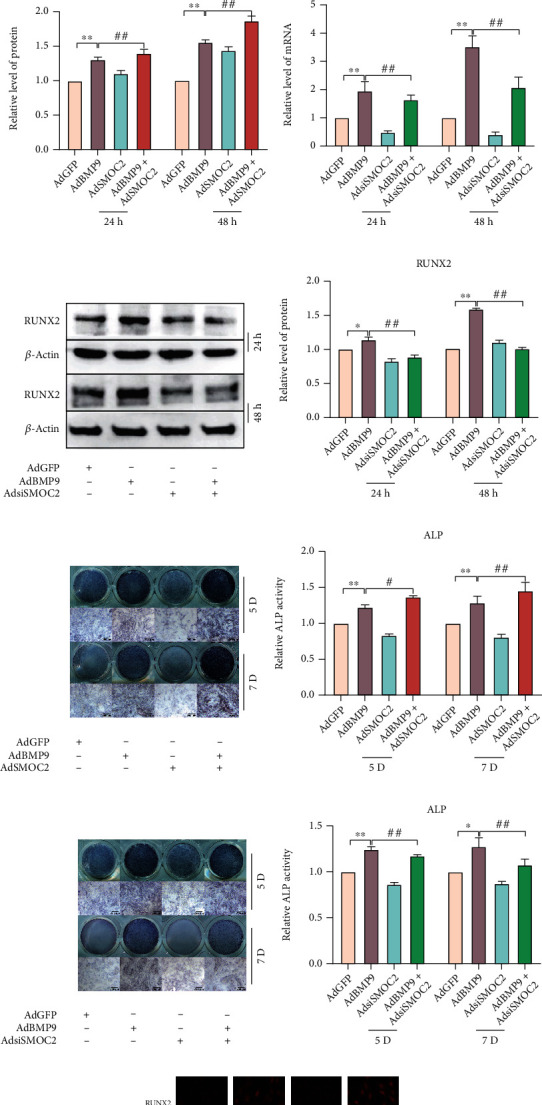
The effect of SMOC2 on BMP9-induced early osteogenic marker levels. (a) Real-time PCR assay results showed the effect of SMOC2 on the mRNA expression of RUNX2 induced by BMP9. (b) Western blot results showed the effect of SMOC2 on the protein expression of RUNX2 induced by BMP9 at 24 h and 48 h. (c) The quantification results of the Western blot results showed the effect of SMOC2 on the protein expression of RUNX2 induced by BMP9. (d) Real-time PCR assay results showed the effect of SMOC2 knockdown on the mRNA expression of RUNX2 induced by BMP9. (e–f) Western blot assay and quantification results showed the effect of SMOC2 knockdown on the protein expression of RUNX2 induced by BMP9 at 24 h and 48 h. (g–h) ALP assay and quantification results showed the effect of overexpression or knockdown of SMOC2 on the activity of ALP induced by BMP9 in MEFs. (k) Immunofluorescence staining assay results showed the effect of SMOC2 on the expression of RUNX2 induced by BMP9. Compared with the control group, ^∗^*p* < 0.05; ^∗∗^*p* < 0.01; compared with the AdBMP9 group, #*p* < 0.05, ##*p* < 0.01.

Next, we investigated whether SMOC2 plays any role in BMP9-induced osteogenic differentiation in MEFs. Subconfluent MEFs were coinfected with AdGFP, AdBMP9, and/or AdSMOC2/AdsiSMOC2. The expression of the recognized early osteogenic marker RUNX2 was measured on Days 1 and 2 after infection. We found that exogenous overexpression of SMOC2 exhibited no significant effect on the expression of RUNX2 by real-time PCR and western blotting analysis; BMP9 increased the expression of RUNX2 in MEFs, which was enhanced when combined with AdSMOC2 infection (Figures 2(a)–2(c)). Moreover, the expression of RUNX2 induced by BMP9 was reduced when combined with AdsiSMOC2 infection (Figures 2(d)–2(f)). Next, we performed an immunofluorescent staining assay for further validation (Figure 2(k)). As expected, similar results were also found with the well-established early osteogenic marker alkaline phosphatase (ALP) on Day 5 and Day 7. When BMP9 expression was fixed, overexpression of SMOC2 significantly induced an increase in ALP activity, while downregulation of SMOC2 reduced ALP activity (Figures 2(g)–2(j)). These data suggest that SMOC2 may potentiate BMP9-induced osteogenic differentiation in MSCs, although SMOC2 alone exerts no substantial osteogenic effects.

### 3.3. Exogenous SMOC2 Expression Enhances BMP-9-Induced Late Osteogenic Marker Expression and Matrix Mineralization

**Figure 3:**
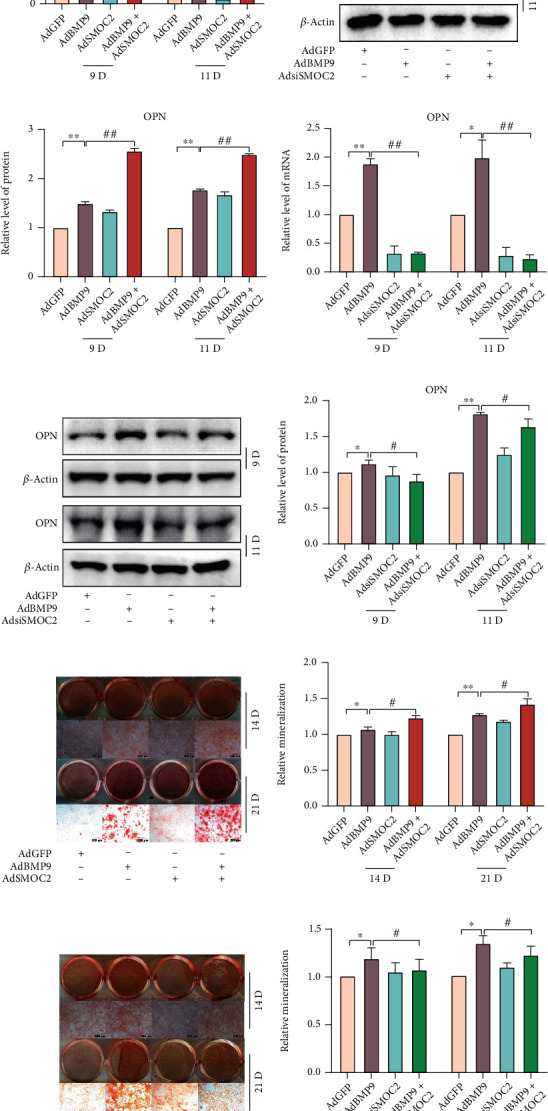
Exogenous SMOC2 expression enhances BMP-9-induced late osteogenic marker expression and matrix mineralization. (a) Real-time PCR assay results showed the effect of SMOC2 on the mRNA expression of OPN promoted by BMP9 in MEFs. (b) Western blot assay results showed the effect of SMOC2 on the protein expression of OPN promoted by BMP9 in MEFs. (c) Quantification of the western blot results showed the effect of SMOC2 on OPN protein expression promoted by BMP9 in MEFs. (d) Real-time PCR assay results showed the effect of SMOC2 knockdown on the mRNA expression of OPN promoted by BMP9 in MEFs. (e) Western blotting assay results showed the effect of SMOC2 knockdown on OPN protein expression induced by BMP9 in MEFs. (f) Quantification of the western blot results showed the effect of SMOC2 on OPN protein expression promoted by BMP9 in MEFs. (g) Alizarin Red S staining results showed the effect of SMOC2 on BMP9-induced mineralization in MEFs. (h) Quantification results of Alizarin Red S staining showed the effect of SMOC2 on BMP9-induced mineralization in MEFs. (i) Alizarin Red S staining results showed the effect of SMOC2 knockdown on BMP9-induced mineralization in MEFs. (j) Quantification results of Alizarin Red S staining showed the effect of SMOC2 knockdown on BMP9-induced mineralization in MEFs. Compared with the control group, ^∗^*p* < 0.05; ∗^∗^*p* < 0.01; compared with the AdBMP9 group, #*p* < 0.05, ##*p* < 0.01.

We further determined the effect of SMOC2 on the BMP-9-induced late stage of osteogenic differentiation. Osteopontin is a well-established marker of late-stage bone formation. We infected MEFs with AdGFP, AdBMP9, and/or AdSMOC2 or AdsiSOMC2. The infected cells were subjected to real-time PCR and western blotting analysis on Days 9 and 11 after infection. We found that exogenous expression of SMOC2 did not exhibit a significant effect on OPN expression, but SMOC2 increased BMP9-induced OPN expression (Figures 3(a)–3(c)). In addition, the inhibition of SMOC2 substantially decreased the mRNA level of OPN induced by BMP9, as well as the protein level of OPN (Figures 3(d)–3(f)). Moreover, we also examined the effect of SMOC2 on BMP9-induced matrix mineralization in MEFs. The mineralization assay showed that mineralized matrix nodules were readily detected in the BMP9-treated group on Day 14 after infection, and the mineralized nodules in the BMP9 combined with SMOC2-treated group were slightly increased compared with those in the group treated with BMP9 only. A similar result was found on Day 21. In addition, the inhibition of SMOC2 decreased the mineralized nodules induced by BMP9 both on Days 14 and 21 after infection (Figures 3(g)–3(j)). These findings strongly suggest that a combination of BMP9 and SMOC2 can promote the BMP9-induced late stage of osteogenic differentiation in vitro, although SMOC2 itself has a very limited ability to induce the late stage of bone formation. However, the detailed mechanism of SMOC2 on the effect of matrix mineralization induced by BMP9 is not clear.

### 3.4. Activation of FAK Signaling in the Enhancement Effect of SMOC2 on BMP9-Induced Osteogenic Differentiation in MEFs

**Figure 4:**
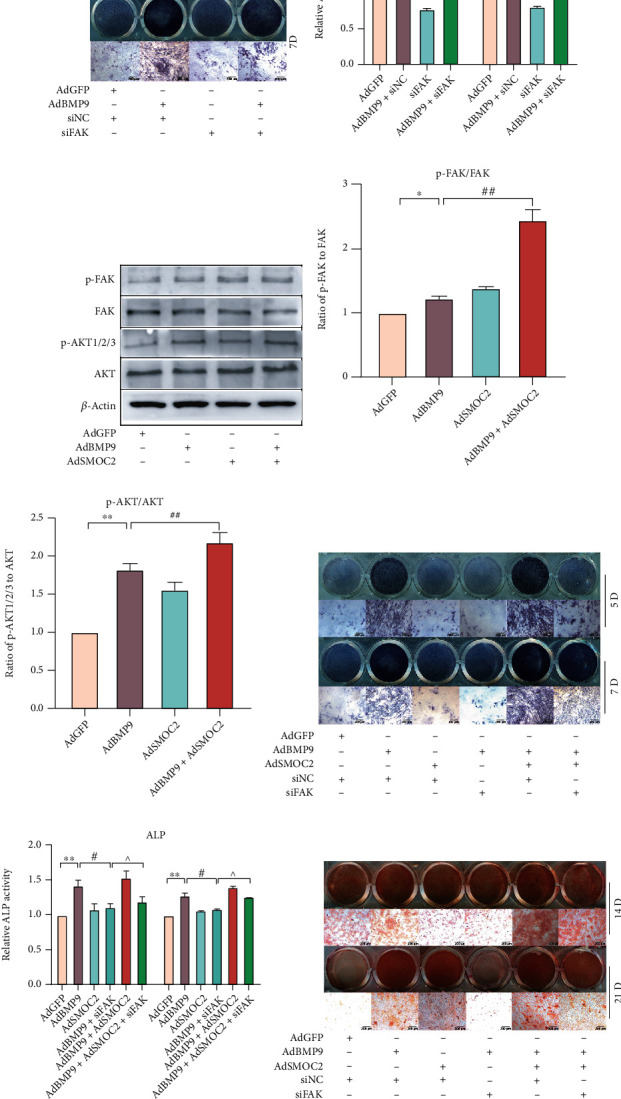
The activation of FAK signaling in the enhancement effect of SMOC2 on BMP9-induced osteogenic differentiation in MEFs. (a) Western blot assay results showed that the levels of p-FAK and FAK were affected by BMP9 in MEFs at 30 h. (b) Quantification of the Western blot results showed that the levels of p-FAK and FAK were affected by BMP9. (c) ALP assay results showed the effect of FAK knockdown on the activity of ALP induced by BMP9 in MEFs. (d) Quantification results of the ALP assay showed the effect of FAK knockdown on the activity of ALP induced by BMP9. (e) Western blot assay results showed the effect of SMOC2 on the expression of p-FAK and p-AKT promoted by BMP9 in MEFs. (f–g) The quantification results of western blotting showed the effect of SMOC2 on the expression of p-FAK and p-AKT promoted by BMP9 in MEFs. (h) ALP assay results showed the reverse effect of SMOC2 on the effect of FAK knockdown on the BMP9-induced activity of ALP. (i) Quantification results of the ALP assay showed the reversal effect of SMOC2 on the effect of FAK knockdown on the BMP9-induced activity of ALP in MEFs. (j) Alizarin Red S staining showed the reversal effect of SMOC2 on the effect of FAK knockdown on BMP9-induced mineralization in MEFs. (k) Quantification results of Alizarin Red S staining showed the effect of SMOC2 on the effect of FAK knockdown on BMP9-induced mineralization in MEFs. Compared with the control group, ^∗^*p* < 0.05; ^∗∗^*p* < 0.01; compared with the AdBMP9 group, #*p* < 0.05, ##*p* < 0.01; compared with the AdBMP9 and siFAK group, ^*p* < 0.05, ^^*p* < 0.01.

Next, we investigated the mechanism by which SMOC2 enhances BMP9-induced osteogenic differentiation of MEFs. Since SMOC2 was found to promote the proliferation of hepatocellular carcinoma cells by enhancing FAK signaling [22], we wondered whether SMOC2 also enhances FAK signaling in MEFs. Interestingly, at 24 h after infection with AdBMP9, the western blotting results showed that BMP9 increased the phosphorylation level of FAK in a dose-dependent manner, which is consistent with the protein level of SMOC2 induced by BMP9 (Figures 4(a)–4(b)). Next, we used siFAK, which targets FAK, for further investigation. The ALP assay showed that siFAK had no effect on ALP activity but substantially inhibited the ALP activity induced by BMP9 on Days 5 and 7 (Figure 4(c)). The difference was statistically significant (Figure 4(d)). Then, we determined whether FAK can be regulated by SMOC2. Western blotting analysis showed that SMOC2 increased not only the endogenous level of p-FAK but also the phosphorylation level of FAK induced by BMP9. Similar results were found for the phosphorylation level of AKT (Figures 4(e)–4(g)). Moreover, while siFAK decreased the ALP activity induced by BMP9, SMOC2 partially reversed this inhibitory effect (Figures 4(h) and 4(i)). Further confirmation was carried out by using the Alizarin Red S staining assay. As expected, we found that SMOC2 partly reversed the inhibitory effect of siFAK on BMP9-induced mineralized matrix nodules in MEFs (Figures 4(j) and 4(k)). These results suggest that siFAK may reduce the enhanced effect of SMOC2 on BMP9-induced osteogenic differentiation by inactivating FAK signaling and that SMOC2 potentiates BMP9-induced osteogenic differentiation through FAK signaling.

### 3.5. SMOC2 Potentiates BMP9-Induced Ectopic Bone Formation in MEFs Implanted In Vivo, which Was Inhibited by Knockdown of FAK

**Figure 5:**
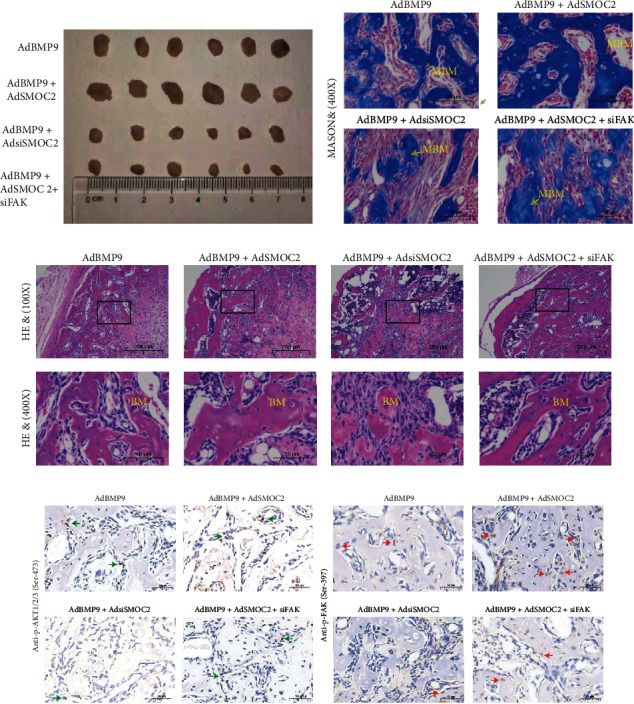
SMOC2 potentiates BMP9-induced ectopic bone formation in MEF implantation in vivo, while knockdown of FAK inhibits bone formation. (a) Macrographic images of ectopic bone mass. MEFs were implanted subcutaneously after infection with the designed adenoviruses. Ectopic osseous masses were retrieved at 4 weeks. Representative images are shown. (b) Masson's trichrome staining results showed the effect of SMOC2 and FAK knockdown on BMP9-induced ectopic bone masses in MEFs (scale bar is 50 *μ*m for the panel). (c) Hematoxylin-eosin (H&E) staining results showed that the osteogenesis ability was affected by SMOC2 and/or FAK knockdown in MEFs (scale bar is 200 *μ*m for upper panel and 50 *μ*m for lower panel). (d) Immunohistochemical staining results showed the expression relationship between p-FAK and p-AKT1/2/3. Ectopic osseous masses were retrieved at 4 weeks. The expression of p-FAK and p-AKT1/2/3 was assessed by immunohistochemical staining analysis. Representative images are shown (scale bar is 50 *μ*m for the panel).

Using our well-established stem cell implantation assay, we next determined the effect of SMOC2 and FAK knockdown on BMP-9-induced ectopic bone formation in vivo. MEFs were treated with AdGFP, AdBMP9, AdSMOC2, AdsiSMOC2, and/or siFAK. Then, the infected cells were implanted subcutaneously into athymic nude mice. After four weeks, the mice were sacrificed, and the bone masses were retrieved (Figure 5(a)). No bone masses were found in the AdGFP or AdSMOC2 group. The largest bone masses were found in the AdBMP9 and AdSMOC2 groups, and the BMP9-transduced cells formed bony masses, which were noticeably larger than those formed in the AdBMP9 and AdsiSMOC2 groups. In addition, the bone masses in the AdBMP9, AdSMOC2, and siFAK groups were much smaller than those in the AdBMP9 + AdSMOC2 group. The H&E staining results showed that BMP9-induced bone formation was enhanced by overexpression of SMOC2, while knockdown of SMOC2 inhibited it. As expected, knockdown of FAK reduced the potentiation effect of SMOC2 on BMP9-induced bone formation. Interestingly, on histologic examination, bone masses formed in the AdBMP9 and AdsiSMOC2 cotransduced cell group showed a significant number of undifferentiated mesenchymal progenitor cells compared with the AdBMP9 group (Figure 5(c)). These results were consistent with those of the Masson's trichrome staining, which showed that SMOC2 significantly augmented BMP9-induced matrix mineralization but that silencing SMOC2 inhibited it. In addition, the effect of SMOC2 on BMP9-induced osteogenesis could be further offset by silencing FAK (Figure 5(b)). The bone mass was also subjected to immunohistochemical staining. The expression of p-FAK and p-AKT1/2/3 was detected in the bone mass in the AdBMP9 and AdBMP9/AdSMOC2 groups, whereas a lower than basal level of staining was observed in the AdBMP9/AdsiSMOC2 group (Figure 5(d)). Collectively, these data strongly suggest that SMOC2 may be an enhancer for BMP9 in the induction of osteogenesis in vivo, which may be mediated by FAK.

### 3.6. The Activation of FAK/AKT Signaling May Be Triggered by Facilitating the Interaction of SMOC2 and Integrin *β*1 in MEFs

**Figure 6:**
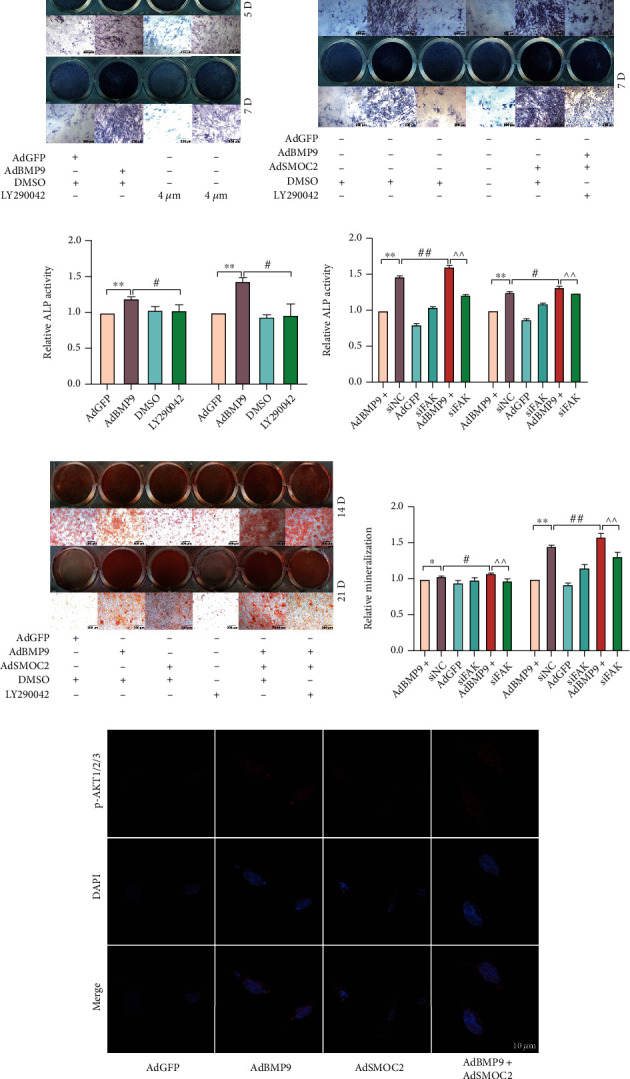
The activation of FAK/AKT signaling may be triggered by facilitating the interaction of SMOC2 and integrin *β*1 in MEFs. (a) Western blot assay results showed the effect of SMOC2 and/or siFAK on the phosphorylation levels of FAK and AKT promoted by BMP9 in MEFs at 30 h. (b–c) Quantification of the western blot results showed the effect of SMOC2 and/or siFAK on the phosphorylation levels of FAK and AKT promoted by BMP9 in MEFs. (d) ALP assay results showed the effect of LY290042 on the activity of ALP induced by BMP9 in MEFs. (e) ALP assay results showed the effect of SMOC2 and/or LY290042 on BMP9-induced ALP activity in MEFs. (f) Quantification results of the ALP assay showed the effect of LY290042 on the activity of ALP induced by BMP9 in MEFs. (g) Quantification results of the ALP assay showed the effect of SMOC2 and/or LY290042 on BMP9-induced ALP activity in MEFs. (h) Alizarin Red S staining showed the effect of SMOC2 and/or LY290042 on BMP9-induced mineralization in MEFs. (i) Quantification results of Alizarin Red S staining showed the effect of SMOC2 and/or Ly294002 on the effect of BMP9-induced mineralization in MEFs. (j) Confocal laser scanning immunofluorescence staining results showed the effect of SMOC2 on the phosphorylation of AKT induced by BMP9 in MEFs at 30 h. (k) ChIP assay shows the enrichment of GTF2I at the promoter region of *s*moc2 in MEFs. (l) Immunoprecipitation assay (IP) results showed that SMOC2 may interact with integrin *β*1 in MEFs. Compared with the control group, ^∗^*p* < 0.05; ^∗∗^*p* < 0.01; compared with the AdBMP9 group, #*p* < 0.05, ##*p* < 0.01; compared with the AdBMP9 and LY290042 group, ^*p* < 0.05, ^^*p* < 0.01.

The data showed that BMP9 increased both the expression of SMOC2 and the phosphorylation level of FAK and that SMOC2 potentiated the phosphorylation level of FAK induced by BMP9. FAK signaling may be implicated in the potentiation of SMOC2 on the osteogenic potential of BMP9. Our western blot results showed that knockdown of FAK inhibited the phosphorylation level of FAK induced by BMP9, while SMOC2 partly reversed this effect (Figures 6(a) and 6(b)). Moreover, knockdown of FAK inhibited the BMP9-induced phosphorylation level of AKT and the phosphorylation level of AKT potentiated by SMOC2 (Figure 6(c)). This result suggested that FAK was implicated in the phosphorylation of AKT induced by BMP9. Since we had already found that knockdown of FAK substantially reduced the ALP activity induced by BMP9, LY294002 was used to prove whether AKT signaling participates in enhancing the effect of SMOC2 on BMP9-induced osteogenic differentiation. As expected, the ALP assay showed that BMP9-induced ALP activity was remarkably inhibited by LY294002 (Figures 6(d)–6(f)). In addition, the BMP9-induced activity of ALP was reduced by LY294002, whereas SMOC2 partially reversed this effect (Figures 6(e) and 6(g)). Further confirmation was carried out by using an Alizarin Red S staining assay (Figures 6(h) and 6(i)). Moreover, confocal microscopy results showed that SMOC2 could increase the phosphorylation of AKT induced by BMP9, which was consistent with our western blot assay results (Figure 6(j)).

**Figure 7:**
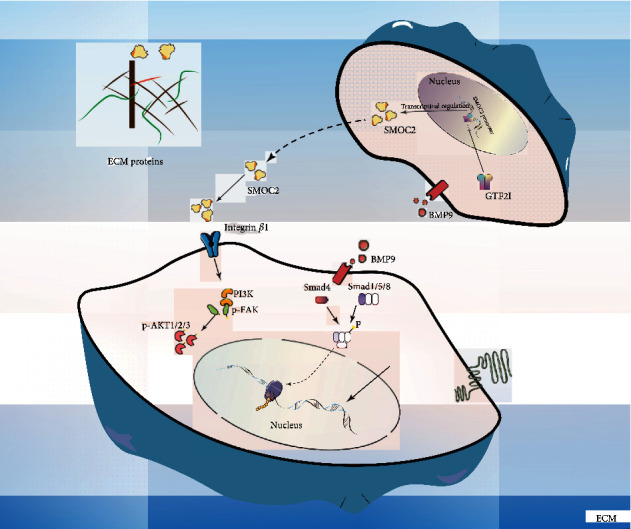
Mechanisms of action of SMOC2 in BMP-induced osteogenic differentiation. The activation of FAK/AKT signaling may be triggered by facilitating the interaction of SMOC2 and integrin *β*1.

It was reported that integrins play an important role in MSC fate determination, and integrin-ECM interactions may activate FAK signaling [23, 24]. Therefore, we employed ChIP and IP assays to elucidate the mechanism by which BMP9 promotes SMOC2 expression and whether SMOC2 enhances FAK signaling by interacting with integrins in MEFs. The results showed that GTF2I was enriched at the promoter region of *s*moc2 and that integrin *β*1 interacted with SMOC2 in BMP9-treated MEFs (Figures 6(k) and 6(l)). This suggests that SMOC2 potentiated BMP9-induced osteogenic differentiation through the enhancement of the FAK/PI3K/AKT pathway, which may be triggered by facilitating the interaction of SMOC2 and integrin *β*1 (Figure 7).

## 4. Discussion

In the process of stem cell fate determination, BMP9 is considered to be one of the most promising osteogenic inducers. It is conceivable that more efficacious bone regeneration may be achieved if BMP9 is administered with other factors, such as matricellular protein SMOC2. In the current study, we investigated the effect of SMOC2 on BMP9-induced bone formation in MSCs. Here, we found that SMOC2 can promote the osteogenic capacity of BMP9 by promoting the activation of FAK, which may be triggered by facilitating the interaction of SMOC2 and integrin *β*1. Additionally, we further demonstrated that GTF2I was recruited to the promoter of *S*MOC2.

In this report, we used BMP9, also known as transforming growth factor 2, as an osteogenic inducer. BMP9 was first found in the genome of the developing mouse liver [25]. Similar to osteogenic inducers such as BMP2, BMP4, and BMP7, BMP9 can induce osteogenic differentiation of MSCs and facilitate bone defect repair [2]. BMP9 normally performs physiological functions through the BMP/Smad pathway. Multiple previous studies have revealed that BMP9 binds to ALK1/2 type I receptors and forms a heterotetrameric complex, after which the intracellular structural domain of BMPR I initiates SMAD1/5/8 phosphorylation. Then, phosphorylated SMAD1/5/8 and SMAD4 combine to form the SMAD1/5/8-SMAD4 complex, which eventually translocates into the nucleus, thereby regulating downstream target genes, such as RUNX2 and OSX. Moreover, BMP9 can also regulate the osteogenic differentiation of a variety of MSCs by affecting multiple signaling pathways or other essential factors, e.g., COX2, retinoic acids, IGF2, and FAK [18, 26–32]. We previously demonstrated that IGF1 and IGF2 could both amplify BMP9-induced MSC differentiation by increasing PI3K/AKT signaling [32, 33]. However, the functional role of BMP9 in regulating the lineage-specific differentiation of MSCs remains to be fully elucidated.

Matricellular proteins are a class of nonstructural ECM proteins that regulate cellular processes and cell-matrix interactions, such as growth factor signaling and cell differentiation [34, 35]. It has been established that various matricellular proteins play key roles in stem cell fate [36]. Through cell-matrix interactions, matricellular proteins guide and regulate not only MSC morphology but also their differentiation, proliferation, and survival. Most matricellular proteins bind to multiple integrins, which allows cells to sense molecular cues and activate downstream signaling [24]. For instance, fibronectin 1 was reported to activate WNT/*β*-catenin signaling to induce osteogenic differentiation via integrin *β*1 interactions [37]. In this study, we observed that matricellular protein SMOC2 was dramatically increased during BMP9-induced differentiation in MEFs (Figure 1(a)), and endogenous expression of SMOC2 was detectable in multiple mesenchymal stem cells (Figure 1(d)), suggesting that SMOC2 may be associated with the osteogenic function of BMP9 in MEFs.

SMOC2, a recently identified matricellular protein that belongs to the SPARC family [38], exists in two isoforms. In humans, SMOCs regulate cell growth factor signaling, cell proliferation, migration, and angiogenesis [39, 40]. In the skeletal system, SMOC2 is involved in the development of bones; for example, mutations in the SMOC2 gene have been linked to canine brachycephaly, and SMOC2 deletion in zebrafish has been linked to craniofacial skeletal dysplasia [41, 42]. In mice, SMOC2 deficiency affects bone repair and results in age-dependent bone loss [43]. In addition, Thomas et al., using SMOC deletion constructs, found that SMOC-∆EC lacks the extracellular calcium binding (EC) domain and inhibits BMP2 signaling, while SMOC-EC (EC domain only) enhances BMP2 signaling, suggesting that SMOC2 could act as an antagonist of BMP signaling and an extender [44]. These differential effects of SMOC2 on the bone skeletal system may be caused by several factors, including the cell types and the cellular microenvironment. However, the effect of SMOC2 on the osteogenic differentiation of multipotent progenitor cells is not clear, and few studies have explored this. More studies should also focus on the interactions of SMOC2 with cell surface receptors to better understand the molecular mechanisms triggered by SMOC2. Recently, Schüler et al. demonstrated that SMOC2 could interact with integrin 1 during aging, resulting in impaired muscle stem cell activity and regeneration [45]. Previous research has also identified several SPARC members as effectors of cell shape, adhesion, and differentiation via integrin *β*1 interaction and signal transmission via integrin linking kinase and/or GSK-3*β* [34, 46, 47]. Based on these findings, we hypothesized that SMOC2 may also regulate BMP9-induced osteogenic differentiation in MEFs via interaction with integrins.

To our knowledge, there are no previous reports on the relationship between SMOC2 and BMP9-induced osteogenic differentiation in MEFs. This study revealed a relationship between SMOC2 and BMP9. BMP9 increased both the transcriptional and translational levels of SMOC2 in MEFs, and overexpression of SMOC2 in turn increased BMP9-induced osteogenic markers, whereas SMOC2 knockdown inhibited this. A similar result was found in vivo, which showed that overexpression of SMOC2 potentiated BMP9-induced bone formation, while SMOC2 knockdown inhibited it. To elucidate the potential mechanism by which SMOC2 contributes to BMP9-induced bone formation, an IP assay was used to show that SMOC2 could interact with integrin *β*1 during BMP9-induced osteogenic differentiation in MEFs (Figure 6(l)), suggesting that SMOC2 may be associated with integrin signaling during stem cell differentiation in MEFs.

To the best of our knowledge, FAK is a cytoplasmic nonreceptor tyrosine kinase that plays an important role in bone health, and FAK activation is decreased in osteoblasts from osteoporotic human bone compared to osteoblasts from normal controls [48]. Several investigations have shown that FAK is an important signaling molecule that coordinates cytoskeletal changes during lineage differentiation [49, 50]. Activation of FAK is regulated by integrins or growth factor receptors on the plasma membrane. Mechanistically, interactions between integrins and ECMs promote FAK dimerization and its autophosphorylation at the Y397 site [23], which then interacts with the regulatory subunit of phosphoinositide 3-kinase (PI3K), thereby leading to the activation of the serine/threonine kinase AKT [51]. In the current study, SMOC2 was found to interact with integrin *β*1 in MEFs, and the important roles of integrins in MSC differentiation have been demonstrated in previous studies. To investigate whether FAK signaling is involved in SMOC2's interaction with integrin *β*1 to regulate BMP9-induced osteogenic differentiation in MEFs, the levels of related proteins were measured. Western blot assays showed that the phosphorylation of FAK at Y397 could be promoted in a BMP9-dependent manner, while BMP9 had no effect on total FAK protein. In addition, knockdown of FAK inhibited BMP9-induced ALP activity (Figures 4(a) and 4(b)), which is consistent with a previous study [52], suggesting that FAK signaling is involved in BMP9-induced osteogenic differentiation. In addition, the phosphorylation of FAK induced by BMP9, as well as the phosphorylation of AKT, could be potentiated by SMOC2 (Figure 4(e)). Knockdown of FAK inhibited the phosphorylation of FAK induced by BMP9, while SMCO2 partly reversed this effect. Furthermore, LY290042 inhibited the ALP activity and mineralized matrix nodules induced by BMP9, whereas SMOC2 partly reversed the inhibitory effect of LY290042. Further analysis also showed that GTF2I is enriched in SMOC2's putative promoter region in MEFs (Figure 6(k)). Therefore, based on these findings, we propose a novel mechanism to elucidate the involvement of SMOC2 on BMP9-induced osteogenic differentiation in MEFs.

In this study, MEFs were derived from the embryonic stage with multiple differentiation potentials, they can differentiate into osteogenesis when stimulated by osteogenic-inducing factors, and can induce the formation of ectopic bone subcutaneously in vivo. Therefore, the MEFs used in our study were mesenchymal stem cells. It has multidirectional differentiation potential and is easier to obtain than BMSC. A previous study reported using an in vitro protocol to reprogram MEFs into functional osteoblast-like cells, which indicates that MEFs can be used as potential cells for bone tissue engineering [53]. Recently, induced pluripotent stem cells-derived MSC (iPSC-MSCs) has been proposed as an alternative resource. iPSCs generated with this pluripotent have numerous applications, including expansion to large scale cell numbers for tissue engineering and the development of cellular therapeutics [54–56]. Here, although MEFs cells derived from embryonic tissues show pluripotency, the exact mechanism remains to be investigated. Meanwhile, the great risk of cell transformation and carcinogenesis from different tissue sources also deserves our attention. We still need to verify SMOC2's role in the extracellular matrix in a wider range of cell lines or tissues.

In conclusion, through bioinformatics techniques and in vivo experiments, we identified a novel function of SMOC2 during the process of BMP9-induced osteogenic differentiation in MEFs. Our experiments suggest that SMOC2 could potentiate BMP9-induced osteogenic differentiation through the FAK/PI3K/AKT pathway, which may be triggered by facilitating the interaction of SMOC2 and integrin *β*1. GTF2I may potentiate the osteogenic ability of BMP9 to upregulate SMOC2, but the exact mechanisms need to be further studied. Collectively, our findings and subsequent research on SMOC2 may provide a new novel target in BMP9-induced osteogenesis of MEFs.

## Figures and Tables

**Table 1:** The primers used for PCR.

Gene	Primer	Sequence (5′⟶3′)
SMOC2	F	CCTGGCCACCTTCTTCTTGT
	R	TTGCATTTCTCGGAGCCTGT

RUNX2	F	GCCAATCCCTAAGTGTGGCT
	R	AACAGAGAGCGAGGGGGTAT

OPN	F	TGCACCCAGATCCTATAGCC
	R	CTCCATCGTCATCATCATCG

*β*-Actin	F	CCACCATGTACCCAGGCATT
	R	CGGACTCATCGTACTCCTGC

SMOC2 (ChIP)	Primer1 F	CGGTCCACATGTTAGTGCCT
	Primer1 R	TCCGAACTTGGAACTCTGGC
	Primer2 F	GATGATGGCTTCCACACCCA
	Primer2 R	ACTTCATCAGGTGGGCTGTG
	Primer3 F	ATCTCCAGAGCAGCAGAGGA
	Primer3 R	AAACCAAGGGGATTTTGCGC

F: forward; R: reverse.

## Data Availability

The RNA-seq data used to support the findings of this study are available from the corresponding author upon request.
